# Does sickness absence history affect work participation after vocational labour market training?

**DOI:** 10.1093/eurpub/ckac131.267

**Published:** 2022-10-25

**Authors:** L Salonen, S Solovieva, A Kauhanen, E Hartikainen, E Viikari-Juntura, T Leinonen

**Affiliations:** 1 Finnish Institute of Occupational Health, Helsinki, Finland; 2 ETLA Economic Research, Helsinki, Finland

## Abstract

**Background:**

Active labour market programmes, such as vocational training, have become widely adopted measures to increase job seekers’ employment opportunities, but evidence of their effectiveness in later employment is mixed. Individuals who enter training differ greatly in their socioeconomic background, health, and labour market history, which can affect the effectiveness of these programmes. There is still uncertainty, for example, on how vocational labour market training works for individuals with prior health problems.

**Methods:**

We use nationally representative Finnish register data on 88,283 individuals aged 25-59 who participated in vocational labour market training in 2008-2013, 23,715 of whom had sickness absence lasting at least 10 weekdays (including Saturday) three years prior to the training and 64,568 of whom did not. To adjust for the differences in sociodemographic and work-related factors between these groups, we will conduct propensity score matching. We will analyze work participation three years before and after the training among those with sickness absence and those without using a difference-in-difference analysis.

**Results:**

Our preliminary results show that before training the work participation rate was similar in those with sickness absence history (58.5 %) and those without (58.1 %). After training, those with a sickness absence history had slightly lower work participation than those without (54.6% versus 59.6 %). The differences in work participation between these groups increased slightly over time after training.

**Conclusions:**

Vocational labour market training does not necessarily work equally well for everyone in terms of enhancing employment, and job seekers with work disability history should be offered either extra support or another type of unemployment service. However, these are our pre-matching results, and they cannot be causally interpreted. Next, we will conduct the propensity score matching and difference-in-difference analysis.

**Key messages:**

• Having a sickness absence history prior to vocational labour market training is associated with lower work participation after training compared to participants without a sickness absence history.

• The main advantage of the study is the use of a quasi-experimental study design to test the effectiveness of vocational training, using rich nationally representative register data with long follow-up.

